# Supplementary figures and images for: Paradoxical effects on voltage-gated Na+ conductance in adrenal chromaffin cells by twin vs single high intensity nanosecond electric pulses
Source: PLoS One. 2020 Jun 9;15(6):e0234114. doi: 10.1371/journal.pone.0234114 (PMC7282663; doi:10.1371/journal.pone.0234114)

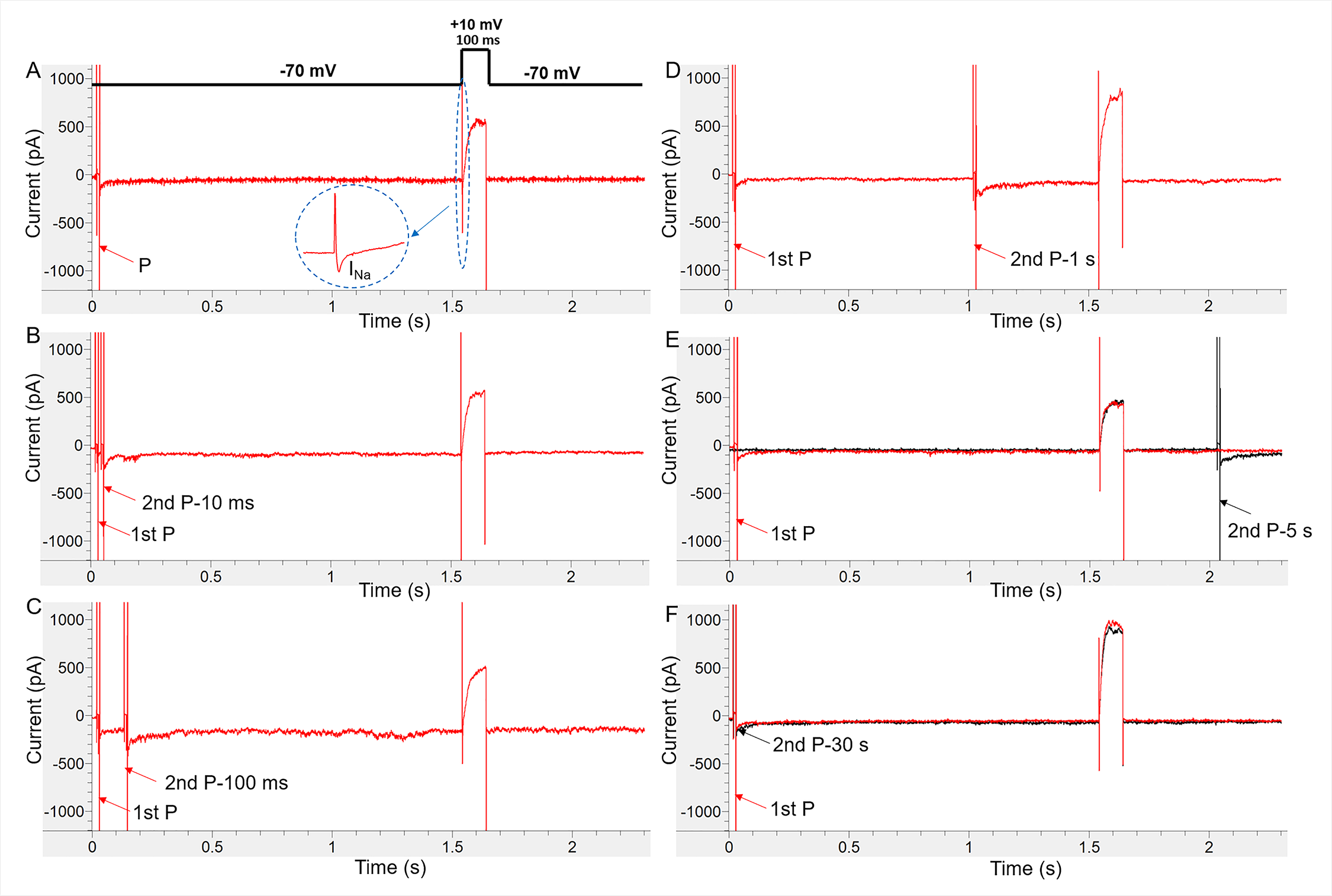

Supplement: S1 Fig — Traces in all panels were from different experiments. The constant voltage step protocol consisted of applying a 100 ms voltage clamp step to +10 mV from a holding potential as shown at the top of panel A. Traces of inward currents were continuously recorded by applying a total of 200 voltage clamp steps, with a 3 s interval between each step. The inset in panel A shows an expanded view of the peak inward current. Representative traces of inward current following exposure of a cell to a single 5 ns pulse (A) or a pulse pair with time interval of 10 ms (B), 100 ms (C), 1 s (D), 5 s (E) or 30 s (F). For panels A thru D, the single pulse or pulse pair (red line) was applied just prior to the 21st voltage clamp step in the sequence to record to current, with an interval of 1.5 s for the single pulse (P) or the first pulse (1st P) of a pulse pair. For the 10 ms (B) 100 ms (C) and 1 s data set (D), the second pulse (2nd P) of a pulse pair interval was applied with an interval of ~ 1.49, ~ 1.4 and ~ 0.5 s, respectively, prior to the application of the voltage clamp step. For panels E and F, because the interval was longer than the membrane recording sweep duration, the membrane current traces from different sweeps were superimposed and displayed by different colors. For the 5 s data set (E), the second pulse (black line) was delivered after the 22nd voltage clamp step with an ~ 2.5 s interval between the second pulse and recording the sodium current recording during the 23rd voltage clamp step (not shown). For the 30 s data set (F), the second pulse interval (black line) was ~ 1.5 s prior to recording the inward current during the 31st voltage clamp step. (TIF) [file pone.0234114.s001.tif]

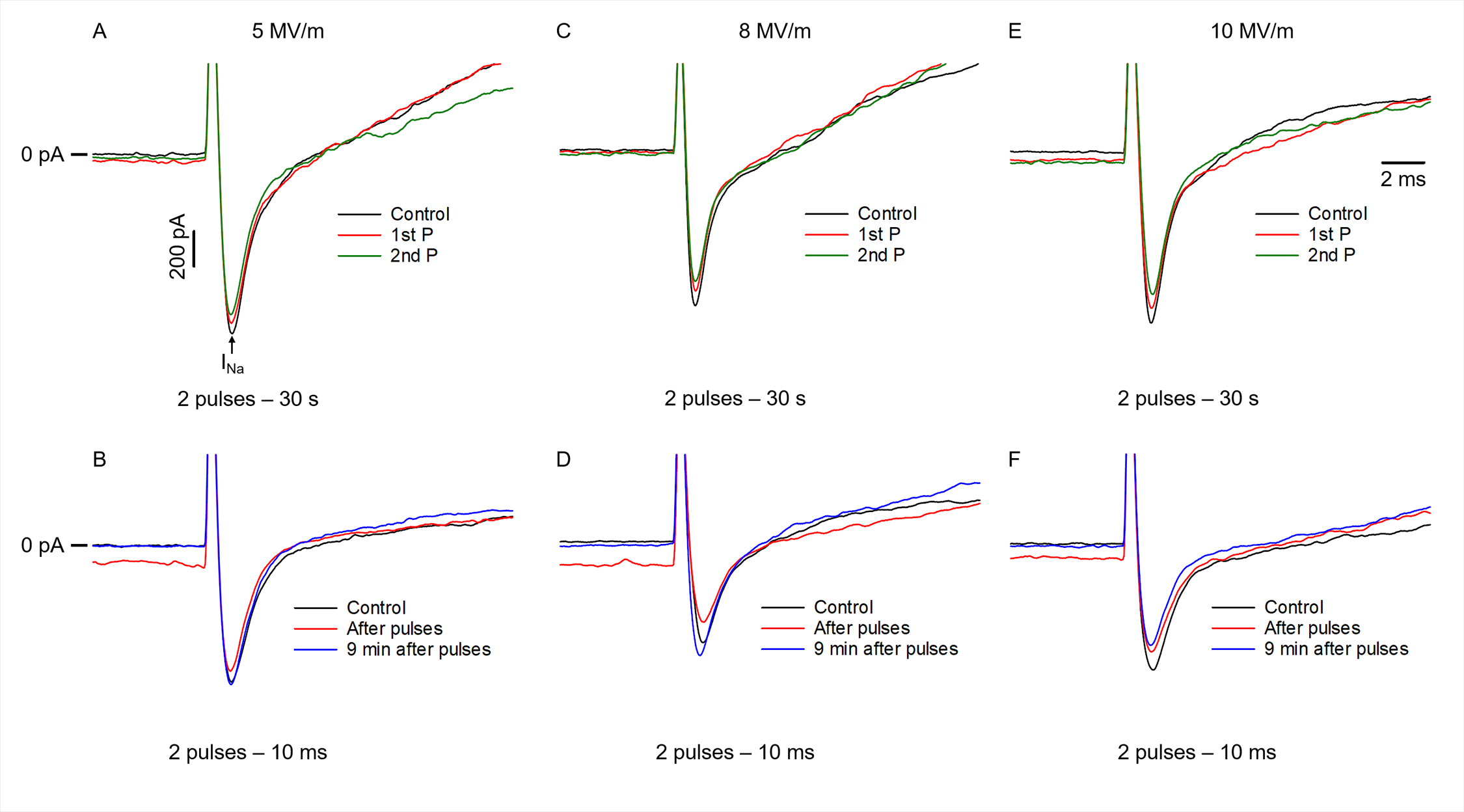

Supplement: S2 Fig — Traces in all panels were from different experiments. INa traces were elicited by voltage clamp steps to +10 mV from a holding potential of -70 mV as described in S1 Fig. Representative traces of INa recorded before, and after the first (1st P) and second pulses (2nd P) at an E-field of 5 MV/m (A), 8 MV/m (C) or 10 MV/m (E), respectively. Representative traces of INa recorded before, immediately after the twin pulses with an interval of 10 ms (After pulses) and 9 min after the application of the twin pulses (9 min after pulses) at an E-field of 5 MV/m (B), 8 MV/m (D) or 10 MV/m (F), respectively. (TIF) [file pone.0234114.s002.tif]
