# Supplementary material for: Paradoxical effects on voltage-gated Na+ conductance in adrenal chromaffin cells by twin vs single high intensity nanosecond electric pulses
Source: PLoS One. 2020 Jun 9;15(6):e0234114. doi: 10.1371/journal.pone.0234114 (PMC7282663; doi:10.1371/journal.pone.0234114)
Supplement: Attachment — Submitted filename: Yang et al 2020 R1 Response to Reviewers_Final.docx [file pone.0234114.s003.docx]

**Reply to Reviewer #1:**

We want to thank the reviewer to have taken his or her precious time to review our manuscript. We also appreciate the reviewer’s fair and constructive criticisms. As will be noted, we have undertaken several series of experiments to address these points and have accordingly revised the manuscript extensively. Figures 1 and 7 are new figures. We also added 2 figures showing raw data traces in the Supporting Information section and 10 references. All changes made to the manuscript are highlighted in red.

1. “This manuscript presents results from experiments to examine the effect of two closely spaced high intensity nanosecond electric pulses on voltage-dependent sodium (Na_V_) current in bovine chromaffin cells. The twin pulses are varied by intensity and interval and clear differences are observed as these parameters are altered. While the effects on the inward current are interesting, there are no definitive experiments that point to a clear mechanism. In addition, there is one result that appears to suggest that Na_V_ channels are not directly affected by these pulses, which is against one of the main conclusions of this work.”

Response:

We agree that our experiments do not point to a clear mechanism but do provide possible avenues to explore in the future. The biggest problem in this field has been and remains that the cellular and molecular effects of these high intensity nanosecond pulses (NEPs) cannot be directly visualized. Investigators have relied on molecular dynamics simulations and experimental strategies yielding indirect but yet valid information on how these pulses affect the lipid bilayer and transmembrane proteins. The patch clamp technique is an invaluable tool as it allows to directly monitor how the function of ion channel protein with great temporal resolution, which is not the case for most transmembrane proteins. Using this approach, cholesterol depletion with methyl-β-cyclodextrin (MβCD), which has profound effects on the biophysical properties of the membrane, dissociated the inhibitory effect of twin NEPs on I_Na_ from their impact on its recovery after the inhibition has taken place. The idea that disruption of lipid rafts by MβCD prevented the recycling of Na_V_ proteins following twin NEPs is certainly not a proof of the actual mechanism but is a key step in laying a foundation for future investigations.

With respect to the second point made by the reviewer, we performed several new series of experiments to address this concern. Our new data show that the rapid inward current was abolished by replacing external Na^+^ with N-Methyl-D-Glucamine^+^ or by bath application of Tetrodotoxin (5 μM; TTX). As new Figure 1 shows from the I-V recorded in the presence of NMDG^+^ or TTX, the current at +10 mV, which was the main voltage step used to probe the time-dependent effects of twin NEPs, was close to 0 pA. This is because the activation of Ca^2+^ and voltage-dependent K^+^ current is much slower than I_Na_. This of course was no longer valid at potentials near the reversal potential as the smaller I_Na_ caused by the reduced driving force for Na^+^ was contaminated by a balance of inward Ca^2+^ (probably very minor as its driving force is also reduced) and outward K^+^ currents. This is evident from the results of new experiments in Figure 7A showing that the inward current was very virtually superimposable across the entire voltage range with the exception of potentials more positive than +50 mV when recorded in normal solution (BSS) or in solutions designed to isolate I_Na_ (Cs^+^ + TEA + Mg^2+^). This valid point made by the reviewer forced us to reexamine whether NEPs influence the steady-state voltage-dependence of I_Na_. We performed new series of experiments under conditions that isolated I_Na_ by suppressing voltage-dependent Ca^2+^ and K^+^ currents (Fig. 8). Finally, and importantly, we also carried out one series of experiments under the latter conditions and found that the results mirrored those previously obtained with BSS (Fig. 7B). Taken together, these results now provide compelling evidence that twin NEPs targeted I_Na_. We want to thank the reviewer again as the addition of these data have considerably strengthened our interpretation and conclusions.

2. “One of the first observations presented is that the Na_V_ current ‘runs down’ with time. The authors speculate that this results from a slow leftward drift in the voltage-dependent gating parameters, which has been observed previously. However, the data presented in their earlier paper (on a single high intensity pulse) argues against such a shift. Figure 2a of that paper shows that peak inward current remains at +10 mV for the entire 15 min recording period. On the other hand, outward currents show a large reduction over the same time period at +50 mV. The slow loss of the ‘N’ shape of the outward current IV suggests that the current actually running down is voltage-dependent calcium (Ca_V_) current, which is well known to express this phenomenon. Since the Na_V_ current has not been isolated in these experiments, it seems that Ca_V_ current rundown is a likely explanation for the slow loss of peak inward current. If this is true, one wonders if these experiments are focused on the wrong current.”

Response:

Figure 2a of Yang et al. (2017) that the reviewer refers to reflects one sample experiment in which I_Na_ didn’t run down. However, on average the current ran down ~ 6% over the course of 10 min (Fig 5a of Yang et al., 2017). Although significant, this rundown was small compared to that seen for the outward current, which we speculated (as alluded by the reviewer) was due to the well-known rundown of Ca^2+^ currents indirectly activating a Ca^2+^-activated K^+^ current. In fact, the experiment shown in Figure 2a of Yang et al. (2017) would instead argue that the rundown of I_Na_ was completely dissociated from that of the outward current as this would have led to an “apparent” significant enhancement of I_Na_, which was not observed despite a large rundown of the outward current over a 15 min period. This argument, combined with the new experiments discussed in point #1 above, again strongly argue in favor of the early inward current solely reflecting the activity of I_Na_ and that its rundown was not due to the rundown of Ca^2+^ and/or K^+^ currents.

3. “One of the most difficult aspects of this work to understand is why isolated Na_V_ current was not studied. This was also the case in the previous publication. The authors argue that peak inward current is primarily comprised of Na_V_ current, but at +10 mV it is clear that Ca_V_, K_V_ and Ca-activated K currents also contribute. If the effect of the nanopulses were larger, the author’s argument for a Na_V_ channel effect would be strengthened. However, the reduction varies between 5-14%, which supports the idea that any of the above-mentioned currents could be mediating these effects. The experiments on isolated Na_V_ currents should be done.”

Response:

Points #2 and #3 above provide strong support that twin NEPs influenced I_Na_ and this was not due to other contaminating conductances. The reason that we had not initially isolated I_Na_ was that the observation of the paradoxical recovery of I_Na_ in response to twin pulses was made during the course of our previous study that led to the publication by Yang et al. (2017). Many of those experiments were therefore performed under similar conditions with normal bath and pipette solutions. We felt confident at the time that the inward current, at least at +10 mV, was a valid reporter for I_Na_. One aspect that we had not emphasized in the first submission of the current article, and we sincerely apologize for this, was the observation by Yang et al. (2017) that the inward current recorded using normal solutions was abolished by replacing external Na^+^ with NMDG^+^, which was confirmed in the present study by repeating those experiments, and by the addition of TTX experiments.

4. “While the majority of data shown are time courses of inward current measured at +10 mV, Figure 6 shows both normalized and relative conductance vs. voltage (GV) curves. Figure 6C shows the relative GV curve, which appears to discount the hypothesis that this is an Na_V_ channel effect. The relative conductance is only reduced at voltages > 0 mV by the twin nanopulses. If the nanopulse effect were due to a reduction in the number of active Na_V_ channels (as proposed by the authors), one would observe a 14% reduction over the entire GV curve, not only at voltages > 0 mV. One possible explanation is that K_V_ current is enhanced by the nanopulse. This idea is supported by the voltage-dependent activation of K_V_ current shown in the previous publication (activation > -10 mV). If this were true, a shift in the reversal potential would likely be observed. Unfortunately, IV curves are not shown, so it not possible to see if such a shift exists. Another problem is that none of the discussed mechanisms can explain the preferential reduction of inward current at voltages > 0 mV. The relative GV curve shown in the previous publication shows a similar preferential effect at depolarized voltages.”

Response:

This is a valid point made by the reviewer, which we have carefully considered. As mentioned in point #1 above, all experiments examining the effects of twin NEPs on the steady-state voltage-dependence and conductance of I_Na_ were repeated in K^+^-free, Cs^+^- and TEA-containing pipette and bath solutions, with Mg^2+^ replacing Ca^2+^ in the bathing solution, to inhibit K^+^ and Ca^2+^ currents. We now show I-Vs for all conditions tested (Fig. 8B), with the addition of another I-V for the so-called “leak” conductance (I_leak_) as previously reported by our group in bovine chromaffin cells (Yoon J, Leblanc N, Zaklit J, Vernier PT, Chatterjee I and Craviso GL. Enhanced monitoring of nanosecond electric pulse-evoked membrane conductance changes in whole-cell patch clamp experiments. *J Membr Biol*. 2016;249:633-644) and hypothesized to be manifest of the process of electronanoporation. The latter I-V was generated in the same conditions described above using a slow voltage ramp protocol to voltage-inactivate Na^+^ channels. It was used to correct the I-V of I_Na_ measured immediately after the application of twin NEPs, but not that recorded 9 min after the pulses since I_leak_ slowly disappeared during that time period. Our data show that the I-Vs were nearly identical with or without this correction, with the exception of voltages near the reversal potential. The correction led to I-V relationships that displayed reversal potentials that were not significantly different for I_Na_ measured prior, immediately after, and 9 min post NEPs. The correction was of course crucial for the conductance calculations shown in Fig. 8D, which rely on normalizing peak current to the driving force (V – E_rev_). These experiments and measurements hopefully have alleviated the reviewer’s concern about that the main effect of twin NEPs was to reduce maximal Na^+^ conductance. The changes are now apparent at potentials > -10 mV. The reason for the apparent lack of effect at -30 and -20 mV probably has to do with the small but not significant leftward shift of the activation curve (~ - 3 mV) and the fact, as pointed out by the reviewer, the magnitude of the effect of twin pulses is small.

5. “In the Discussion section, the authors describe the possibility that the nanopulses produce new pathways for ions to permeate the cells as leak currents (not voltage-dependent). However, do data are presented on the holding current. If such ‘leak’ pathways were generated, there should be an increase in holding current if those pathways were Na^+^ or Ca^2+^ selective, or if they were non-selective for cations. If such leak pathways were K^+^ selective, holding current may not be significantly altered at -70 mV (the holding potential). Such an increase in K^+^ leak could be observed by making brief, strong hyperpolarizing steps (-120 mV perhaps) to look for increased holding current at a voltage with a driving force strong enough to observe K^+^ flux.”

Response:

This point is partially discussed in point #4 above. We now provide direct measurements of the voltage-dependence of this leak current, which displayed slight inward rectification and reversed near 0 mV suggesting a non-selective cation conductance. Measuring this current allowed us to make a correction for peak I_Na_ measurements, which led to a reversal potential that was unaffected by NEPs. As evident from Fig. 8B, I_leak_ was minuscule (only a few pA) relative to I_Na_ at +10 mV and was not responsible for the major effects of twin NEPs on the inward current. We are currently working on another project investigating the biophysical properties of this current, which will be the subject of another manuscript in preparation. We believe that this “basal” conductance may be, at least in part, involved in mediating the membrane depolarization triggered by NEPs in this cell type. Finally, we added a section in the Discussion reporting the results of an interesting study by Rems et al. (bioRxiv. 2019. doi: doi.org/10.1101/838474) that we only recently became aware. This paper describes molecular dynamics simulations suggesting that NEPs may directly alter the properties of voltage-gated ion channels including Na^+^ channels by rendering them voltage-independent and promoting the emergence of alternate hydrophilic pathways near the voltage sensor domains of the channel. Although theoretical, the study has relevance to our experiments revealing the appearance of I_leak_ and a reduction in Na^+^ conductance in response to NEPs.

6. “One thing missing from the figures is the actual ionic currents. The currents at +10 mV should be shown with the time courses for the three times used for the GV curves, namely control, peak reduction of inward current and at 9 min. It is also important that a biophysical analysis be done on the currents. At least the rise time of the inward current and information on the outward current(s) needs to be added. Otherwise the reader is not able to independently assess the author’s conclusions.”

Response:

Thank you for this excellent suggestion. We added families of traces generated from an I-V protocol for a typical experiment showing the inhibition of I_Na_ by twin NEPs at 8 MV/m separated by a 1 s interval (Fig. 8B). We also added examples of traces in S1 and S2 figs. We will reserve to perform a thorough biophysical analysis of how NEPs influence I_Na_ in a future study in which all experiments will be carried out under conditions isolating this current. We think adding information on outward K^+^ currents will be confusing to the reader as this study is focused on the effects of NEPs on I_Na_.

7. “The authors state that the reduction of current due to the nanopulses is instantaneous, but does not appear to be true. There is a clear time course to the current reduction, which is easily observed for the larger nanopulse effects. Such a time course should be investigated as it is possible that clues to the mechanism could be gleaned from such an analysis.”

Response:

Thank you for this valid point. We have changed the text to reflect that the effects of NEPs were rapid but not instantaneous as the reviewer correctly pointed out. We agree that the time-dependent changes after the application of NEPs are interesting and should be investigated. However, we feel that this manuscript is already bulky, and it is not immediately clear what new experiments could be devised to study the impact of such changes. We will certainly consider examining this interesting observation in the future.

8. “The methyl-β-cyclodextrin experiment is interesting, but impossible to interpret without more information. The currents need to be shown so that the reader can see if outward currents are also affected. Here the authors also admit that there is a role for KV currents in shaping the inward current, since they hypothesize that a shift in the reversal potential is due to the increased influence of these currents. Even though the reversal potential is measured at a more depolarized voltage (~+40 mV) than +10 mV, this highlights the problems with interpretation of voltage clamp data from currents that are not isolated.”

Response:

This issue has already been addressed in previous points above.

**Reply to Reviewer #2:**

We want to thank the reviewer for a careful review of our manuscript. We have undertaken several series of experiments to address several points raised by the reviewers and have extensively revised the manuscript in accordance with all comments and suggestions. Figures 1 and 7 are new figures. We also added 2 figures showing raw data traces in the Supporting Information section and 10 references. All changes made to the manuscript are highlighted in red.

1. “All data does not appear fully available. Only mean values are presented within the manuscript and figures. Please make full data available either as supporting information or in a public repository.”

Response:

We now provide original Na current traces in Figure 8, and S1 and S2 figs in the Supplementary Information section. If accepted for publication, all raw data will be made available upon request.

2. “No primary experimental evidence linking caveolae to the results is presented, and methyl-b-cyclodextrin universally depletes cholesterol within cells. Cholesterol impacts numerous membrane properties and functions in addition to fusion, trafficking and recycling, such as bilayer fluidity and the second messenger molecule, as mentioned in the Discussion. Therefore, it is recommended to reword line 368 (perhaps “investigate” instead of “determine”), and to remove “capable of disrupting caveolae integrity” in line 506.”

Response:

Thank you for this valid point. We have revised the sentences in the manuscript as suggested.

3. “Regarding the influence of cholesterol depletion on cellular responses to 10 ns and 600 ns electric pulses, consider citing this 2016 publication by Cantu et al. (<https://doi.org/10.1016/j.bbamem.2016.07.006>).”

Response:

This reference has been cited and discussed.

4. “Capitalization of the title is inconsistent with the others in Reference 47 (lines 710-713).”

Response:

We have corrected this reference.

**Reply to Reviewer #3:**

We want to thank the reviewer for his or her positive comments and a careful and fair evaluation of our manuscript. We have undertaken several series of experiments to address several points raised by the reviewers and have extensively revised the manuscript in accordance with all comments and suggestions. Figures 1 and 7 are new figures. We also added 2 figures showing raw data traces in the Supporting Information section and 10 references. All changes made to the manuscript are highlighted in red.

Major Comments:

1. “The manuscript by Yang et al., describes the effects of dual high intensity pulses on voltage-gated sodium channel currents in adrenal chromaffin cells. There are interesting findings on the recovery of the sodium channel current in response to dual rather than single NEPs and the effects of depletion of membrane cholesterol. The manuscript is generally well written but requires clarification on the methodology and statistical tests used which could determine whether some conclusions are in agreement with the data presented.

   1) Throughout the manuscript, the authors conclude that there are differences in the magnitude of inhibition and / or recovery of voltage-gated Na^+^ channel currents in chromaffin cells exposed to dual NEPs in comparison to those exposed to a single NEP. However, with the exception of the data presented in Figure 6, there is no mention of which statistical tests were used to conclude that the responses were significantly different. Could the authors provide the results from the statistical tests in the manuscript text and figures?”

Response:

Thank you for this excellent point. We now provide the results of statistical tests, both in the text and figure legends, throughout the manuscript as recommended.

2. “The authors concluded that there was an additive effect on inhibition only when the interval between the NEPs exceeded 100ms. With regards to this, could the authors clarify a couple of points in the methodology? In the methods, the authors state that a single NEP or pair of NEPs was applied between the 20th and 21st voltage steps with an interval of 0.5s between the time the NEP was delivered and recording of currents.

“a) Did the voltage step depolarizations to +10mV every 3s continue during the interval between the two NEPs? In figures 1B, 3B and 4B (all 30s NEP intervals) there look to be data points that indicate the step depolarizations were continued in between exposure to the two NEPs.”

“b) Does the 0.5 s interval between the time the NEP was delivered refer to the first NEP? If there is an interval of 0.5 s between the first NEP of the pair and the subsequent step depolarization to record I_Na_, is the second NEP exposure after the 21st voltage step for any cells in which the interval between two NEPs is 1s or greater? In addition, is the time between exposure to the 2nd NEP and the next step depolarization to record I_Na_ equal or different for each dual NEP exposure interval? For example, in the 10 ms data is the 2nd NEP-voltage step interval ~ 490 ms and in the 1s data the same interval ~ 2.5 s?

It would aid the reader if the authors could depict this protocol, indicating after which voltage step the second NEP exposure occurred and the time interval between NEP exposure and the next step depolarization.”

Response:

We apologize for this typo. Recording of the sodium current was initiated during the 21^st^ voltage clamp step after an interval of 1.5 s (instead of 0.5 s as wrongly stated) following the delivery of a single NEP or the first of the two NEPs for twin pulses. We edited the Methods to clarify this. The voltage clamp step depolarizations to +10 mV applied at a frequency of 1 step every 3 s continued during the interval between the two NEPs. We added also added S1 fig to illustrate the timing of delivery of the 21^st^ voltage clamp step relative to that of a single NEP or 1^st^ NEP of a pulse pair. The exception to this rule was that for the 5 s and 30 s delay data sets, the 2^nd^ NEP was respectively applied prior to the 23^rd^ and 31^st^ voltage clamp steps. This is due to the fact that I_Na_ recorded after the second pulse occurred on a subsequent recording sweep. In these two cases, membrane recording traces were superimposed and depicted by two distinct colors.

3. “c) If the 2^nd^ NEP-voltage step time interval is unequal, does varying the interval between a single NEP, of different electric field strengths, and a voltage step to record I_Na_ affect the voltage-gated sodium channel inhibition observed?”

Response:

This is a very good point. We tested the influence of different electric field strengths. As showed in the manuscript, a single pulse applied at an E-field amplitude of 5 MV/m resulted in a rapid decrease of ~4% in peak I_Na_ followed by a much slower exponential decline to a level that was ~89% of the initial level after 10 min. Increasing the E-field amplitude to 8 or 10 MV/m caused a twofold greater inhibitory effect on peak I_Na_. There was no further decline in I_Na_ over the course of 10 min, indicating that the effect of the pulse on I_Na_ had saturated at the higher E-field amplitudes of 8 and 10 MV/m. These effects were also reported in our previous paper that was published in PLOS ONE in 2017 (L. Yang *et al*. PLoS One. 2017;12(7):e0181002). For the benefit of the reviewer, we carried out additional experiments in which we varied the interval between the time the NEP was delivered and the recording of sodium current initiated. We found very similar results whether I_Na_ was recorded after or 0.5 or 1.5 s after a single pulse as shown in the figure below. We did not explore other intervals since the focus of this study was to compare the effects of single and twin NEPs on I_Na_.

Minor Comments:

1. “In figures 1-4 is the same control or 1 pulse data set used in all panels? Could the authors state in the figure legends if this is the case.”

Response:

The Control data are the same for Figures 1-4 and we now provide this information in the legends as suggested.

2. “Legend for Figure 1 – I think page 10 line 209 should read voltage step to +10 mV rather than -10 mV.”

Response:

This typo was corrected. Thank you.

3. “It would be useful to the reader to see some representative sodium current traces.”

Response:

We now provide representative traces in Figure 8 as well as in S1 and S2 figs.

4. “Could the authors provide more detail in the methods on the double pulse protocol used to determine steady state inactivation including conditioning pulse duration, test pulse duration and voltage step, interval between pulses. It would also be useful to the reader to provide a schematic of this protocol and the steady state activation protocol alongside the relevant data sets.”

Response:

We now provide this information in the Methods. Illustrations of the protocols used are also shown in relevant figures.

5) PLOS does not permit references to data shown. In the instance where this refers to the reversal potential, it may be helpful for the readers to see the IV curves that were used to determine steady state activation and conductance rather than remove the reference to this data.

Response:

We are now showing I-V curves in Figure 8.
