# Supplementary material for: Paradoxical effects on voltage-gated Na+ conductance in adrenal chromaffin cells by twin vs single high intensity nanosecond electric pulses
Source: PLoS One. 2020 Jun 9;15(6):e0234114. doi: 10.1371/journal.pone.0234114 (PMC7282663; doi:10.1371/journal.pone.0234114)
Supplement: Attachment — Submitted filename: Yang et al 2020 R2 Response to Reviewer #1 Final.docx [file pone.0234114.s004.docx]

**Reply to Reviewer #1:**

We want to thank the reviewer to have again taken the time to review the revised version of our manuscript. We are very appreciative of the reviewer’s overall satisfaction with our revisions. We have edited the manuscript in accordance with all points raised. Below is a point by point response to the new points raised by the reviewer:

1. “The double NEPs present several different effects over a single NEP. First, the size of the Na_V_ current reduction is generally larger, as one would expect. Second, there is a recovery of Na_V_ current amplitude, but only if the two pulses are ≤ 100 ms apart. Interestingly, this recovery is actually an over-recovery, since the current amplitude is even larger than the current would be under control conditions, at least for twin NEPs 10 ms apart and < 10 MV/m. Indeed, the lack of full recovery with 10 MV/m NEPs at 10 ms interval suggests a different mechanism involved to prevent recovery. The data are very interesting. The problem is that the Discussion does clearly present these ideas. The speculated mechanisms involved in the initial reduction of current are presented, but the ‘paradoxical’ recovery is not. It would be helpful to the reader if the authors would re-organize the Discussion to talk about the Na_V_ current inhibition by NEPs, including the possible mechanisms, and then the paradoxical recovery, again including possible mechanisms. Further, the enhancement of the current beyond that in control (at 9 min) needs a mechanistic discussion. The recovery is discussed, but that discussion comes before the presentation of the mechanisms for the Na_V_ current inhibition, and therefore there is an absence of mechanisms for the recovery.”

Response:

We would like to thank the reviewer for suggesting a reorganization of the Discussion (Point #1 below), which we have carefully undertaken. We think it now flows better and makes our conclusions stronger. While relooking at it, we realized that our data were indeed discussed in a manner that diluted the main findings and conclusions. We now first discuss the inhibition of I_Na_ by twin NEPs, followed by a separate section about the recovery, both including possible mechanisms. A lot of it was done by shuffling paragraphs. However, we also expanded the section about the recovery of I_Na_ to address the reviewer’s point below about the impact of an acute exposure to MβCD.

2. “One experiment that needs to be added is the effect of acutely applied MβCD on the Na_V_ current. The authors show the effect of a 30 min MβCD pretreatment and conclude that the associated effects are consistent with lipid raft involvement, but this conclusion is only valid if the acute application shows no or significantly smaller effects. It is generally agreed that the 30 min incubation period is required for the disruption of lipid rafts by MβCD.”

Response:

This is a valid criticism but unfortunately, we have been on lockdown since March 18 and partial reopening is scheduled for May 30^th^, per the order of the Governor of Nevada. So, it is currently not possible for us to carry out the series of experiments you are suggesting. The latter date could be extended depending on the state of the pandemics in Nevada. An additional delay of several weeks to reopen our labs under very strict restrictions is to be expected, which would push a resubmission by several more weeks if not months. To circumvent this, we attempted to find other publications that possibly investigated acute effect of MβCD on ion channels. We were unable to find a publication that examined the time course of effects of MβCD on ionic currents. All papers that we found used a preincubation ranging from 5 min up to several hours. This is the text that was added in that section to address your point:

“Whether this effect was the result of a direct acute blocking effect of MβCD on Na^+^ channels, cholesterol depletion *per se* and/or lipid raft disruption is unclear as time-dependent effects of the agent on I_Na_ were not investigated. However, distinguishing between these alternative possibilities would be difficult to interpret because in one study, a 5 min preincubation was sufficient to deplete membrane cholesterol, disrupt lipid rafts and increase Ca^2+^-activated Cl^-^ currents in vascular smooth muscle cells [63]. In most studies, a preincubation with this agent for 15 min or more (30 min in our study), is a standard procedure to deplete membrane cholesterol and eliminate caveolae in cells in which these membrane structures are found [19, 56, 69-72].”

Other comments:

1. “Figure 1. Why is there apparently no Ca_V_ current when NMDG was used to replace Na^+^? Is there a small inward current in TTX that could be attributed to Ca_V_ channels? Clarification would be helpful.”

Response:

We have previously recorded L-type Ca^2+^ currents in these cells (unpublished data) under conditions in which Na^+^ channels were voltage-inactivated and K^+^ channels were inhibited with Cs^+^ and TEA. These currents were smaller than I_Na_ (~ 125 pA at 0 mV instead of 300-700 pA) but more importantly they activated much more slowly, which explains the lack of contamination by these currents in our peak I_Na_ measurements. In Figure 1 specifically, K^+^ channels were not blocked and overwhelmed Ca^2+^ currents. In fact, a large fraction of the outward current recorded around the peak of the I-V relationship for I_Na_ is produced by Ca^2+^-activated K^+^ channels stimulated by Ca^2+^ entry through Ca^2+^ channels as previously reported by our group in the same journal (Yang L, Craviso GL, Vernier PT, Chatterjee I and Leblanc N. Nanosecond electric pulses differentially affect inward and outward currents in patch clamped adrenal chromaffin cells. *PLoS One*. 2017;12:e0181002). These too activated much more slowly, which explains why no evidence of net inward current was apparent when external Na^+^ was replaced with NMDG^+^ or I_Na_ was blocked with TTX.

2. “Figure 6 legend. For the panels showing recovery, the measurement was taken at 9 min after the NEPs, but that time is not mentioned in the figure legend. It is mentioned in the text but should be added to the legend. In this figure, there is no mention of significant differences. It is not clear if the any of the presented valued in each of the graphs are different from one another.”

Response:

We added the timing of the measurements (9 min) for the recovery of I_Na_ in the figure legend of Figure 6 as the reviewer suggested. In terms of statistical analyses performed, this was a concern of Reviewer #3 during the review of the initial version of our manuscript. The results of statistical tests, including P values, were all presented for figures preceding Figure 6. Since Figure 6 is a summary figure essentially presenting the same data to better illustrate the phenomena studied, we felt it was unnecessary to re-add this information for this particular figure.

3. “Line 367. It is stated that K-gluconate was replaced with aspartate, which is not true. The replacement compound was actually Cs-aspartate, at least that is what I could glean from the Methods section. This needs to be corrected.”

Response:

This was corrected. Thank you.

4. “The description of the experiments measuring the leak current need to be clarified. Were these experiments done in the same cells used for the Na_V_ current measurements? It is also not clear why figure 8 presents discrete points for the leak measurement instead of a continuous line, as would be recorded from a ramp. If these points were measured from the ramp, it should be clear how the measurement was done. For example, were a range of values averaged for each point? If so, how wide was the voltage range covered?”

Response:

The information concerning how the leak current was measured was already in the legend of figure 8 and still is:

“I_leak_ was measured in separate experiments using a voltage ramp protocol and K^+^-free external solution containing 20 µM nitrendipine as described in the Methods.”

It was also mentioned in the text (underlined) that the current was measured in separate experiments and that a ramp was used to measure it:

“To determine the impact of this basal conductance on peak I_Na_ measurements_,_ I_leak_ was estimated in separate experiments using K^+^-free pipette and bath solutions containing Cs^+^ and TEA, which were used to suppress voltage-gated K^+^ channels, and 20 μM nitrendipine applied to the bath to inhibit voltage-gated Ca^2+^ channels [18]. A 2 s voltage ramp protocol spanning from ‒70 to +80 mV was used to generate I-V relationships before and after the application of twin pulses with a 1 s interval at E-field of 8 MV/m. Voltage-gated Na^+^ channels were voltage-inactivated by this slow voltage ramp protocol.”

A sentence was added to indicate specifically the voltage range at each potential during the ramp that was used to measure the leak current:

“Leak current measurements were performed by averaging the current during the voltage ramp for a range spanning ± 0.5 mV at each target potential (used for peak I_Na_ measurements).”

5. “The conclusion seems to focus solely on the single NEP, since it describes only the Na_V_ inhibition. There needs to be discussion here of the recovery as well. In addition, there needs to be discussion of the potential selectivity of the NEPs, since the focus here is on pain management. It seems very likely that other Na_V_ channels will also be affected, which would impact other sensory axons.”

Response:

We are not sure what the reviewer is referring to because we did talk about both the inhibition and recovery of I_Na_ in response to twin NEPs:

“The results of this study have shown that I_Na_ in excitable adrenal chromaffin cells can be differentially modulated by a single versus a pair of 5 ns pulses, with the differential effect of the pulse pair depending on both the time interval between the two pulses and the E-field amplitude. Of particular significance is that a pulse pair at 5 MV/m having a very short interpulse interval (10 ms) can not only attenuate the inhibitory effect of a single pulse on I_Na_ but also lead to a paradoxical progressive recovery of the inward current. The enhanced recovery of I_Na_ disappeared at longer interpulse intervals, an effect accentuated at a higher E-field.”

With respect to the last segment of your point, we used pain as an example of how these pulses could be used as novel non-invasive tools to modulate function. We added the following text to broaden this potential therapeutic application:

“It is also possible that other classes of Na_V_ subunits might be similarly or differentially influenced by these pulses, which could affect the excitability of other types of neurons in both the central and peripheral nervous systems. To address this possibility, future experiments will be to investigate the effects of NEPs on I_Na_ in mouse adrenal chromaffin cells that is mediated by both Na_V_1.4 and Na_V_1.7 channels.”
